# Impact of prolonged isoflurane or ketamine–xylazine anesthesia with or without buprenorphine and oxygen on mouse vitals and immune responses

**DOI:** 10.1038/s41684-025-01614-4

**Published:** 2025-09-18

**Authors:** Tommaso Virgilio, Irene Latino, Chiara Pizzichetti, Kamil Chahine, Arianna Capucetti, Carlotta Detotto, Alessandra Bergadano, Santiago F.Gonzalez

**Affiliations:** 1 Institute for Research in Biomedicine, Università della Svizzera italiana, Bellinzona, Switzerland; 2 Graduate School for Cellular and Biomedical Sciences, University of Bern, Bern, Switzerland; 3 Experimental Animal Center, University of Bern, Bern, Switzerland

**Keywords:** Immunology, Imaging the immune system

## Abstract

Anesthesia is indispensable for minimizing stress during invasive procedures. However, anesthesia can induce undesired effects on the organism and its physiological homeostasis. In experiments involving animals, these consequences may reduce animal welfare and alter experimental outcomes. Moreover, most of the studies characterizing the effects of murine anesthetic protocols on animal welfare do not explore the impact of prolonged anesthesia for procedures lasting more than 2 h. Here we investigated the effect of prolonged anesthesia on vital parameters and immune responses to vaccination, comparing isoflurane (Iso), ketamine–xylazine (KX), and KX with oxygen supplementation (KXO_2_), in the presence or absence of buprenorphine (BPP). KX induced hypoxia and 100% mortality, which were prevented by oxygen supplementation (KXO_2_) and were not associated with BPP. By contrast, Iso induced safe and fast induction and recovery. Furthermore, we investigated the effects of these protocols on the immune responses and motility of immune cells following vaccination. The results showed that KX reduced immune cell numbers and increased cell death, complemented by elevated levels of the inflammatory proteins IL-6 and IFNγ. In addition, KX altered the motility patterns of T cells, B cells and neutrophils, potentially influenced by hypoxia. Conversely, Iso exhibited fewer immune artifacts, regardless of BPP. These findings highlight the importance of evaluating anesthesia protocols with respect to animal welfare and experimental reproducibility, especially for immunological studies. Oxygen supplementation emerged as an important refinement to mitigate hypoxia in KX. Iso showed superior safety and fewer artifacts compared with KX and KXO_2_, demonstrating its suitability for immunological research and intravital microscopy.

## Abstract (web summary)

The study reveals that long-term anesthesia with ketamine–xylazine can induce hypoxia and mortality in mice, whereas oxygen supplementation mitigates these effects. As an alternative, isoflurane showed superior safety and fewer effects on the immune system.

## Main

Anesthesia, implemented with analgesia, is essential for inducing unconsciousness and antinociception during invasive procedures in humans and animals to minimize stress. In addition, anesthesia is critical for enhancing procedural efficacy and promoting optimal outcomes in clinical and research settings. However, despite its pivotal role, anesthesia might induce undesired effects on physiological systems.

Inhaled anesthetics are commonly used and considered safe for humans^1^ and laboratory animals^2^. Nevertheless, they do not typically provide intrinsic analgesia. For this reason, when applied for surgery, gaseous anesthetics such as isoflurane (Iso) need to be combined with analgesic agents, such as opioids or nonsteroidal anti-inflammatory drugs, depending on the requirements of the model^2^. Conversely, injectable protocols generally provide hypnosis and antinociception, often achieved by a combination of multiple drugs, while reporting more severe adverse effects than gaseous protocols^2^. For instance, despite being a standard and widely used protocol in mice, the combination of ketamine and xylazine (KX) induces severe side effects, including bradycardia and hypoxia^3^. Because of hypoxia, this anesthetic protocol reduces the safety for animals, leading to a dose-dependent increase in mortality^4^. Importantly, oxygen supplementation has been reported to prevent hypoxia after a single administration of different doses of KX^3^. Furthermore, procedures longer than 2 h require redosing, which might affect the vital parameters of the mouse, as a surgical procedure per se might do. In addition, it is not known how oxygen supplementation in this protocol performs in comparison with standard Iso anesthesia.

Moreover, mouse anesthetics have been observed to alter the immune system, with a potential impact not only on animal welfare but also on experimental readouts. For example, ketamine has been associated with an anti-inflammatory response, blocking the release of cytokines^5,6^ or suppressing Th17^7^ and natural killer (NK)^8^ responses. Controversially, other authors observed a pro-inflammatory response induced by exacerbation of TLR-4 signaling^9^ after ketamine administration. These properties lead to serious consequences, such as an increased risk of postoperative septicemia in mice^10^. Similarly, Iso has also been associated with altered immune responses, including the downregulation of neuroinflammation^11,12^, reduced phagocytosis of pathogens^13^ and decreased NF-κβ signaling^14^.

Despite the relevance of cellular dynamics in the lymphoid organs, the effect of Iso and KX on the motility of immune cells has not been extensively explored. This is of relevance because the dynamic behavior (that is, motility patterns and their biological meaning) of immune cells is a key element for understanding the mechanisms of action of immunity^15^. For example, Iso has been reported to promote the migratory capacity of bone marrow^16^ and microglial cells^17^. Furthermore, oxygenation is pivotal to cellular motility^18^, which is especially relevant considering the hypoxic effect of KX. Therefore, understanding how these anesthetics affect all the previously mentioned parameters is important not only for animal welfare but also for the reproducibility and validity of the experimental results of immune studies. According to the 3Rs principles, improving reproducibility will reduce the number of animals needed to reach significant results^19^.

In this study, we compared the effect of commonly used murine anesthetic protocols, namely Iso and KX, on mouse vital parameters and immunity. We studied the murine immune functionality by measuring the numbers of all the major immune cell types, their release of inflammatory proteins and their dynamic behavior in the lymph node upon stimulation with an influenza vaccine. In addition, we investigated how oxygenation (quantified by pulse oxymetry) and opioid analgesia (buprenorphine, BPP) modulate the effects of KX and Iso.

## Results

### Efficacy and safety of anesthetic protocols

**Fig. 1:**
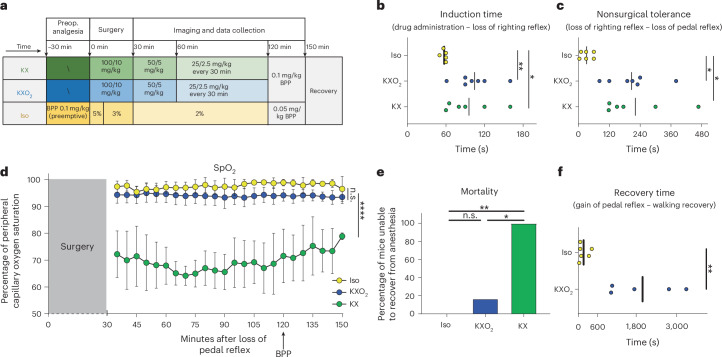
Effect of the anesthetic protocols on mouse vital parameters. **a**, A schematic representation of the three anesthetic protocols. Surgery included the preparation of the pLN for intravital microscopy. Preop., preoperative. **b**,**c**, Induction time, defined as the time passing from drug administration to loss of righting reflex (**b**), and nonsurgical tolerance, defined as the time passing from loss of righting reflex to loss of pedal reflex (**c**). **d**, Oxygen saturation over time. The arrow indicates the time of BPP administration in all the three groups. **e**, Mortality rate for each group indicated as the percentage of mice unable to recover from anesthesia over the total number of mice anesthetized. **f**, Recovery time, defined as the time passing from the gain of pedal reflex to the recovery of the entire walking capacity. In **b**, **c** and **f**, lines and points indicate mean and individual values, respectively. In **d** and **e**, *n* = 6. In **d**, circles indicate the mean value and lines indicate the standard deviation. *P ≤ 0.05, **P < 0.01, ****P < 0.0001. n.s., not significant.

We evaluated the efficacy and safety of three commonly used anesthetic protocols in mice, namely Iso, KX and KX combined with additional oxygen (KXO_2_). We initially designed the protocols of administration of these drugs (dose and frequency) to maintain surgical tolerance for a hypothetical survival experiment lasting two hours and a half, which is the average duration of an intravital imaging experiment using the popliteal lymph node (pLN) surgical model (Fig. 1a). BPP is a potent opioid analgesic with few side effects and is commonly included in Iso anesthesia protocols^2^. In these cases, BPP is administered preemptively to bypass the lack of analgesia. By contrast, in KX protocols, which provide intrinsic analgesia, BPP can be administered 30 min before recovery. The administration protocols were based on established practices in the case of Iso, while we adjusted the redosing of KX (Supplementary Fig. 1a) to achieve uninterrupted surgical anesthesia depth (Supplementary Fig. 1b) as previously described^4^. Importantly, core body temperature was maintained constant in the 37–38.5 °C interval for the entire duration of each experiment. We observed that induction time (Fig. 1b) and nonsurgical tolerance time (Fig. 1c) were significantly shorter in the Iso group compared with KX (*P* = 0.0108 and 0.0112) and KXO_2_ (*P* = 0.0013 and 0.0151). Next, we monitored the oxygen saturation (SpO_2_) of the anesthetized animals over the time of image acquisition (150 min), observing that the KX protocol induced a significant hypoxic state (*P* < 0.0001) during the entire duration of the experiment (Fig. 1d). Conversely, the supplementation of oxygen (FiO_2_ = 1) by face mask rescued the SpO_2_ of the KXO_2_ group to physiological levels^20^, similar to the Iso group (Fig. 1d, *P* = 0.2856). Importantly, the prolonged hypoxia contributed to the 100% mortality rate in KX anesthesia in comparison with Iso (*P* = 0.0043) and KXO_2_ (*P* = 0.0288), showing 0% and 16% mortality, respectively (Fig. 1e).

We hypothesized that cardiac depression induced death in the KX group. We verified this hypothesis by observing that the respiratory rate (RR) (Supplementary Fig. 1c) and the heart rate (HR) (Supplementary Fig. 1d) of KX-anesthetized animals dramatically decreased in the last phase of the recording period. Notably, KX and KXO_2_ induced prolonged bradycardia in comparison with Iso (Supplementary Fig. 1d, *P* < 0.0001). Finally, we measured the recovery time, observing that Iso presented significantly faster recovery than KXO_2_ (Fig. 1f, *P* = 0.0043).

### Effect of BPP on Iso, KX and KXO_2_ protocols

**Fig. 2:**
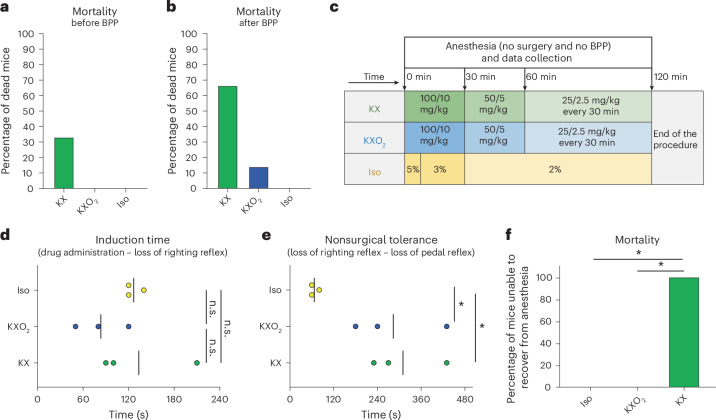
Impact of BPP on Iso, KX and KXO_2_ protocols. **a**,**b**, Mortality rate is described as the percentage of mice unable to recover from anesthesia over the total number of mice anesthetized, before (**a**) and after (**b**) BPP administration. **c**, A schematic representation of the experimental design to assess the effect of BPP in each of the three protocols. No surgery was performed in this experiment. **d**, Induction time, defined as the time passing from drug administration to loss of righting reflex, in all three groups without BPP administration. **e**, Nonsurgical tolerance, defined as the time passing from loss of righting reflex to loss of pedal reflex, in all three groups without BPP administration. **f**, Mortality rate in the three groups without BPP administration. In **a**, **b** and **f**, bars indicate the percentage of events with respect to the total number of animals forming each group (*n* = 3). In **d** and **e**, lines and points indicate mean and individual values, respectively.

Considering that the cardiovascular arrest in the KX group occurred in the last minutes of the procedure, following the injection of BPP, which might induce cardiovascular depression, we hypothesized a correlation between BPP administration and KX mortality. To support this hypothesis, we observed that only 30% of the KX-related mortality occurred before BPP injection (Fig. 2a), while the remaining 70% and all the KXO_2_ cases happened after (Fig. 2b).

To study in more detail the effects of BPP on the protocols under investigation, we measured vital parameters in animals anesthetized with Iso, KX or KXO_2_ without BPP. In this experiment, no surgery was performed to circumvent the use of BPP (Fig. 2c). The absence of preemptive BPP prolonged the induction time of Iso, which was in this case similar to KX and KXO_2_ (Fig. 2d), but not the nonsurgical tolerance time (Fig. 2e, *P* = 0.0457 and 0.0167 for Iso compared with KXO_2_ and KX, respectively). Moreover, the KX group presented 100% mortality, contrasting with the 0% mortality of KXO_2_ and Iso (*P* = 0.0429), even in the absence of BPP (Fig. 2f). Such observation was associated with a prolonged hypoxic state (Supplementary Fig. 2a, *P* < 0.0001), as previously observed, but no drop in RR (Supplementary Fig. 2b) and HR (Supplementary Fig. 2c). These results suggested that both hypoxia and mortality in the KX group were due to the anesthetic protocol itself, and not to BPP administration.

### Effect of the anesthetic protocols on the immune cell dynamics on the lymph node

**Fig. 3:**
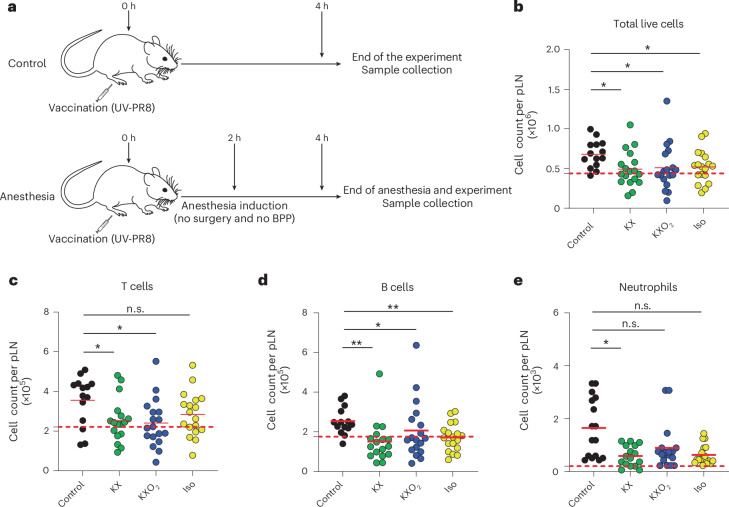
Anesthesia alters the number of immune cells in the lymph node of vaccinated mice. **a**, A schematic representation of the experimental design for flow cytometry experiments. Owing to the absence of surgical stimulation, anesthesia was performed without BPP, following the protocol described in Fig. 2c. UV-PR8 stands for inactivated influenza virus A/Puerto Rico/8/34, used as a vaccine model. **b**–**e**, The number of total live cells (**b**), T cells (**c**), B cells (**d**) and neutrophils (**e**) in the pLN of vaccinated and anesthetized mice in comparison with unanesthetized controls, measured by flow cytometry. Circles and red lines represent individual and mean values, respectively. The control black group is formed by vaccinated, not anesthetized animals. The dashed red bars indicate the average value of not vaccinated and not anesthetized animals.

To study the effect of anesthesia on the immune cell dynamics in the lymph node, we evaluated the immune responses following a model of vaccination with an ultraviolet (UV)-inactivated influenza A/Puerto Rico/8/34 virus (UV-PR8). We quantified by flow cytometry the total number of live cells in the pLN of mice that where anesthetized 2 h after vaccination. In this experiment, mice were not subjected to surgery, and BPP was not implemented in any protocol to avoid unnecessary biases (Fig. 3a). This approach showed a significant reduction in the percentage of living cells following any of the anesthetic protocols in comparison with unanesthetized controls (Fig. 3b, *P* = 0.0101, 0.0223 and 0.0336 for control compared with KX, KXO_2_ and Iso, respectively). Moreover, the reduction of living cells was associated with increased cell death in the KX group in contrast to Iso anesthesia (Supplementary Fig. 3a, *P* = 0.0178).

To characterize the effect of the anesthetic protocols on the individual immune cell types, we quantified the number of immune cells divided by population using flow cytometry. First, we observed that T cells, the most abundant cell type in the pLN, were significantly reduced in the KX (*P* = 0.0222) and KXO_2_ (*P* = 0.0355) groups but not in Iso, in comparison with unanesthetized animals (Fig. 3c). A similar profile was also observed in NK cells (Supplementary Fig. 3b) and monocytes (Supplementary Fig. 3c), for which KX (*P* = 0.0084 and 0.0153) and KXO_2_ (*P* = 0.0118 and 0.0481) but not Iso induced a decrease in the total number of immune cells compared to unanesthetized controls. By contrast, B cells decreased in number in all three anesthetic protocols (Fig. 3d, *P* = 0.0010, 0.0450 and 0.0027 for KX, KXO_2_ and Iso, respectively) compared with unanesthetized controls. Despite an apparent trend in the reduction in all three protocols, neutrophils showed a statistically significant lower number only in the KX group (Fig. 3e, *P* = 0.0446) compared with unanesthetized controls. Among all the cell types under investigation, only macrophages (Supplementary Fig. 3d) and dendritic cells (DCs), classified into CD11b^+^ (Supplementary Fig. 3e) and CD11b^−^ (Supplementary Fig. 3f), were not altered by any of the three anesthetic protocols compared with unanesthetized controls.

### Influence of the anesthetic protocol on the local inflammation

**Fig. 4:**
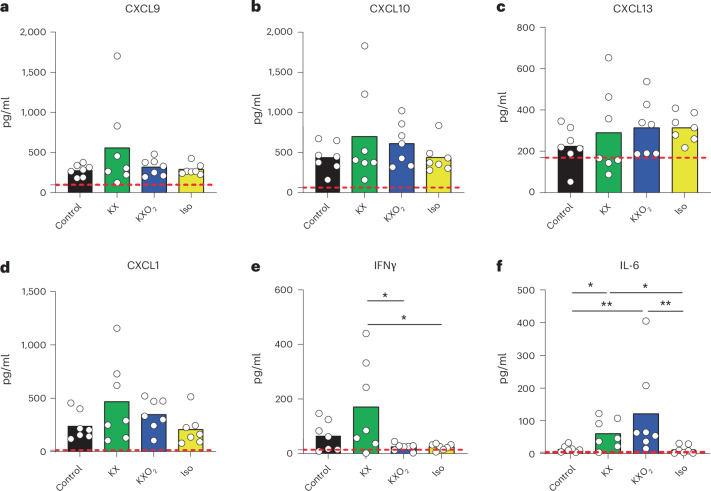
Influence of anesthetic protocols on the release of inflammatory cytokines. **a**–**f**, Quantification of the concentration of the inflammatory proteins CXCL9 (**a**), CXCL10 (**b**), CXCL13 (**c**), CXCL1 (**d**), IFNγ (**e**) and IL-6 (**f**) in the pLN supernatant of vaccinated and anesthetized mice in comparison with unanesthetized controls, measured by flow cytometry. Circles and bars represent individual and mean values, respectively. The control black group is formed by vaccinated, not anesthetized animals. The dashed red line indicates the average value of not vaccinated and not anesthetized animals.

Considering the involvement of inflammatory cytokines and chemokines in the recruitment of leukocytes, we hypothesized that the previously observed differences in the numbers of immune cells might be associated with an altered profile of secretion of these molecules. To investigate this hypothesis, we measured the concentrations of 13 chemokines in the pLN supernatant of vaccinated and anesthetized mice in comparison with not anesthetized controls, following the same experimental design described for flow cytometry in Fig. 3a. The results showed no significant differences between the three anesthetics and the controls for any of the chemokines under study, including CXCL9 (Fig. 4a), CXCL10 (Fig. 4b), CXCL13 (Fig. 4c) and CXCL1 (Fig. 4d), which are among the most relevant chemokines controlling recruitment and homing of T cells, B cells and neutrophils, respectively. In addition, the levels of the other tested chemokines, including CXCL5 (Supplementary Fig. 4a), CCL2 (Supplementary Fig. 4b), CCL3 (Supplementary Fig. 4c), CCL4 (Supplementary Fig. 4d), CCL5 (Supplementary Fig. 4e), CCL11 (Supplementary Fig. 4f), CCL17 (Supplementary Fig. 4g), CCL20 (Supplementary Fig. 4h) and CCL22 (Supplementary Fig. 4i), also did not differ among the experimental groups and the controls. Consequently, we measured the concentration of inflammatory cytokines in the pLN of vaccinated and anesthetized mice. Among them, we observed a significant upregulation of interferon-γ (IFNγ) in the KX group in comparison with KXO_2_ (*P* = 0.0169) and Iso (*P* = 0.0173) (Fig. 4e). In addition, the levels of the inflammatory cytokine interleukin-6 were elevated in both KX and KXO_2_ in comparison with Iso (*P* = 0.0175 and 0.0023) and the control (*P* = 0.0262 and 0.0023) groups (Fig. 4f). Beyond these cytokines, no significant alterations in the concentration of interleukin-1α (Supplementary Fig. 4j), interleukin-1β (Supplementary Fig. 4k), interleukin-10 (Supplementary Fig. 4l), interleukin-12p70 (Supplementary Fig. 4m), interleukin-17A (Supplementary Fig. 4n), interleukin-23 (Supplementary Fig. 4o), interleukin-27 (Supplementary Fig. 4p), tumor necrosis factor (Supplementary Fig. 4q), granulocyte-macrophage colony-stimulating factor (Supplementary Fig. 4r) and interferon-β (Supplementary Fig. 4s) were detected in any group.

### Dynamic behavior of neutrophils, T cells and B cells during anesthesia

**Fig. 5:**
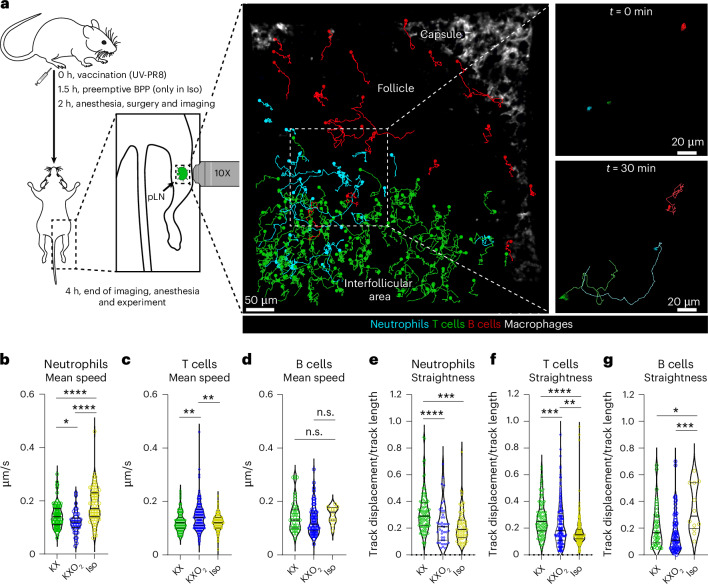
Dynamic behavior of neutrophils, T cells and B cells after anesthesia. **a**, A schematic representation of the experimental design for the 2P-IVM experiments and data generation. **b**–**d**, Quantification of the mean speed, measured in micrometers per second, of neutrophils (**b**), T cells (**c**) and B cells (**d**). **e**–**g**, Quantification of the straightness, measured as the ratio between track displacement and track length, of neutrophils (**e**), T cells (**f**) and B cells (**g**). Within the violin plots, circles indicate individual values, while lines indicate mean and quartiles of distribution. ***P < 0.001.

To study if the anesthetic protocols under investigation also altered the motility of the major immune cells, we analyzed by two-photon intravital microscopy (2P-IVM) the dynamic behavior (that is, the motility patterns in time) of fluorescently labeled T cells, B cells and neutrophils in the pLN of vaccinated and anesthetized mice (Fig. 5a). Considering that 2P-IVM requires surgical preparation of animals for image acquisition, we had to include preemptive analgesia by BPP in the Iso group. We then in vivo imaged immune cells for 2 h in animals anesthetized with each protocol separately and performed manual tracking of neutrophils (Supplementary Movie 1), T cells (Supplementary Movie 2) and B cells (Supplementary Movie 3), independently. We then extracted several parameters related to the motility of these cells, including speed (mean speed of each cell over the time of acquisition) and straightness. The latter is defined as the ratio between cell track displacement and track length and describes how much a cell moves in a specific direction. By comparing the mean speed of each cell under the different anesthetic regimens, we observed that neutrophils moved significantly faster in the Iso group in comparison with the other two groups (*P* < 0.0001), and faster in the KX group in comparison with the KXO_2_ group (Fig. 5b, *P* = 0.0236). In addition, T cells showed a higher mean speed in the KXO_2_ group than in the KX (*P* = 0.0027) and Iso (Fig. 5c, *P* = 0.0020) groups, while B cells did not present any significant difference in mean speed between the three groups (Fig. 5d). We next observed significant differences in the straightness displayed by neutrophils (Fig. 5e) and T cells (Fig. 5f) in the KX group compared with the KXO_2_ group (*P* = 0.0007 and 0.0003, respectively) and Iso (*P* < 0.0001). Furthermore, T cell straightness was observed to be higher in KXO_2_ in comparison with Iso (*P* = 0.0017). Conversely, B cells moved with higher straightness in the Iso group in comparison with the other two groups (Fig. 5f, *P* = 0.0006 for KXO_2_ and 0.0129 for KX).

Considering that preemptive BPP was necessary for 2P-IVM in the Iso group due to surgery, we wondered if analgesia affected the results of Iso. However, it is not possible to perform surgery without analgesia. Therefore, we could not quantify the motility of cells without BPP in this group. Nevertheless, to examine if BPP determines any generic effect on immunity in the Iso group, we investigated the immune cell numbers by flow cytometry in Iso + BPP in comparison with controls (Iso without BPP) and the hypothetical alternative analgesic drug lidocaine (Lido) (Iso + Lido) (Supplementary Fig. 5a). The results showed that BPP did not significantly alter the total number of live cells (Supplementary Fig. 5b), nor did most of the immune subsets, including T cells (Supplementary Fig. 5c), B cells (Supplementary Fig. 5d), NK cells (Supplementary Fig. 5e), monocytes (Supplementary Fig. 5f) and CD11b^+^ DCs (Supplementary Fig. 5g). In all groups, BPP had a smaller effect than Lido, which, in contrast, significantly reduced the total number of live cells (*P* = 0.0187), T cells (*P* = 0.0350), B cells (*P* = 0.0171), NK cells (*P* = 0.0402), monocytes (*P* = 0.0190) and CD11b^+^ DCs (*P* = 0.0485) compared with Iso alone. In addition, neither BPP nor Lido altered the number of CD11b^−^ DCs (Supplementary Fig. 5h) and neutrophils (Supplementary Fig. 5i) compared with Iso alone. By contrast, macrophages were the only cells affected by BPP (*P* = 0.0094), showing a similar reduction of numbers also in Lido (Supplementary Fig. 5j, *P* = 0.0346) compared with Iso alone.

## Discussion

In this study, we investigated the effects of prolonged (2.5 h) KX and Iso anesthesia on mouse vital parameters and immune response. Our first results highlighted a concerning 100% mortality rate in animals repeatedly dosed with KX for long-term maintenance, primarily attributed to hypoxia associated with severe bradycardia, leading to a decrease in tissue oxygen delivery (DO_2_). This finding aligns with previous studies demonstrating that single doses of KX or other different ketamine combinations induce a significant hypoxic state^21,22^. However, single administrations of injectable anesthesia have been shown to induce lethal hypoxia only in particular cases, such as in aged mice, showing 15.4% mortality after KX injection^23^. Similarly to our results, a previous report highlighted significant mortality rates in mice upon consecutive redosing of KX in 30-min intervals^4^. This difference might be associated with amplified hypoxic damage in repeated doses in comparison with single injections. This cumulative effect appears critical for survival only with short-interval redosing, as repeated injections every 3–4 days caused no lethality, as previously shown^24^.

Furthermore, our results suggest that BPP contributes to this morbidity. In fact, BPP analgesia in the KX group probably amplified cardiovascular depression. Moreover, the reduction of the mortality rate from 16% to 0% in KXO_2_ when removing BPP further corroborates this hypothesis. However, the 100% mortality in KX without BPP demonstrates that BPP is not the leading cause of death in these animals. Instead, the prolonged hypoxia appeared to be the pathophysiological event leading to death, probably associated with KX accumulation. It is possible that hypoxia enhances bradycardia and its related reduction of DO_2_ to the tissues and, consequently, mortality. In support of this hypothesis, Choi and colleagues recently demonstrated that xylazine and opioids synergize in inducing brain hypoxia, leading to overdose deaths in a rat model^25^. Importantly, also in rats, the administration of oxygen increased the safety of KX and improved postoperative survival^26^, aligning with the complete prevention of anesthesia-induced hypoxia observed in our murine model. While oxygen supplementation emerged as a relevant refinement to mitigate hypoxia and the related mortality, some authors have suggested that redosing ketamine alone, and not xylazine, for prolonged anesthesia limits the accumulation of anesthetic^27^. This approach is based on the different pharmacokinetic of these two drugs, with ketamine being shorter lasting than xylazine^4^. Furthermore, xylazine, as an adrenergic α2 agonist, is responsible for cardiovascular depression in KX and similar protocols^4^. In fact, repeated administrations of KX can cause redistribution of these drugs in the fat tissue and other anatomical compartments, leading to overdosing^28^. A common strategy to avoid overdosing is to readminister drugs when mice regain some reflexes (for example, pedal) rather than at fixed times. However, this strategy has questionable implications for welfare, as the recovery of reflexes might indicate inadequate surgical depth, with potential intraoperative nociception or even consciousness. In addition, we could not use lower KX doses or redosing concentrations, because they represented suboptimal anesthetic depth in our model, in association with positive withdrawal and eyelid reflexes. Overall, we observed the superior effectiveness and safety of Iso compared with KX and KXO_2_ due to its minor impact on HR, its rapid metabolism and its significantly faster induction and recovery time, regardless of BPP administration. In fact, although RR and HR in the Iso + BPP group were lower than with Iso alone, no side effects or mortality were observed, indicating reasonable safety margins.

Beyond the consequences of the anesthetic protocols on the animal’s vital parameters and survival, we observed notable effects also on the immune response. Interestingly, the reduction in immune cell numbers was accompanied, but not completely explained, by an increase in dead cell numbers. This increase suggests that other mechanisms might cooperate with hypoxia and cell death in reducing immune cell numbers. One of them might be a reduction in cell proliferation. To support this speculation, Cai and colleagues previously demonstrated that Iso could suppress the proliferation of colorectal cancer cells^29^. However, a similar effect has not yet been demonstrated on immune cells. In addition, the timeframe of our experiment (4 h) might be too short for a substantial response on cell proliferation. Another hypothesis is that anesthesia could alter the recruitment of immune cells to the lymph node. Despite this idea not being supported by the unaltered profile of chemokines, which are major controllers of cell recruitment and homing into organs, there are other possible explanations for this theory. For example, a recent study showed that several combinations of anesthetic drugs affect the contractility of the lymphatic vessels, leading to up to 17-fold changes in the contractility frequency in comparison with awake control mice^22^. Even though lymphatic vessels are not the standard route of recruitment of immune cells to the lymph nodes, their contractility is essential to maintain a constant lymph flow and efficient functionality of the lymphatic and immune systems^22^. In addition, xylazine has a major vasoconstriction activity^30–33^. Thus, we cannot exclude that these anesthetics could alter the homing process of the immune cells via the blood vessels. Some of these unexplored effects seemed to be associated only with KX, regardless of oxygen saturation, as, for example, NK cell and monocyte numbers were not altered by Iso.

Despite stable chemokine levels, significant increases in the inflammatory proteins, interleukin-6 (IL-6) and IFNγ, were noted under KX anesthesia compared to unanesthetized controls and the Iso group. In the case of IFNγ, this modification was prevented by oxygen administration, consistently with previous results highlighting hypoxia as a main inducer of IFNγ release by T cells^34^, mainly via upregulation of HIF-1α^35^. Interestingly, propofol, another standard drug for general anesthesia in humans and other species, has also been reported to induce IFNγ release by NK cells in mice, but hypoxia was not investigated in this case^36^. By contrast, the upregulation of IL-6 was not prevented in the KXO_2_ group, suggesting a different drug-related mechanism of induction, not involving hypoxia. Interestingly, IL-6 upregulation following KX has also been previously observed in rats in a model of skin damage^37^. These results are relevant as they show the critical role that both IFNγ and IL-6 play in several immunological processes, such as the responses to vaccines^38^, infections^39^ and cancer^40,41^. Thus, the use of KX might introduce artifacts that have a substantial impact on a broad spectrum of immunological studies.

Cellular motility and dynamic behavior also differed significantly between anesthetic groups. We speculate that these results are the consequence of a combination of the elevated concentrations of inflammatory proteins and the hypoxic or toxic effects of the drugs. In fact, neutrophils showed a different straightness in the KX group in comparison with KXO_2_ and Iso, which is in line with the recognized effect of hypoxia in activating these cells^42^. By contrast, the behavior of T cells seemed to be influenced by the cytokine profile. In fact, the lower straightness of T cells in Iso compared with KX and KXO_2_ is in accordance with a previous report showing that IL-6, upregulated in these anesthetics, enhances the motility of CD4 T cells^43^. Similarly, IFNγ has also been reported to increase T cell motility^44^, potentially explaining why KX showed higher straightness than KXO_2_. On the contrary, B cells presented lower dynamic activity in KX and KXO_2_ than in Iso. This observation could be explained by the fact that IL-6, upregulated in KX and KXO_2_, induces antibody production and B cell proliferation^45^, two static processes usually associated with a stop in B cell movements^46^. Another theory for the altered motility patterns of immune cells is that anesthetic drugs might induce remodeling of the cytoskeleton, a phenomenon previously observed, for example, in breast cancer cells upon Lido treatment^47^. This is particularly relevant considering that Lido can be used to provide analgesia in protocols with insufficient analgesic power, such as Iso. Interestingly, our data show that BPP did not significantly alter the number of immune cells (except macrophages) in comparison with Iso alone, with possibly lower artificial effects on immunity than Lido. However, this comparison was not exhaustive in our investigation owing to the different administration routes, doses and timings of BPP and Lido. One limitation of our approach is the lack of negative controls (no anesthesia and/or no analgesia) for two-photon imaging, as surgical anesthesia is necessary for these imaging procedures. Despite this limitation, considering all the previous results on animal vital parameters, cell numbers and release of the inflammatory protein, we speculate that Iso, regardless of BPP implementation, could be the closest protocol to nonanesthetic physiological conditions, also concerning cellular motility.

In conclusion, our findings suggest that KX induces significant alterations in mouse vital parameters and artifacts in the immune responses to vaccination, which can, in some instances, be prevented by oxygen administration. Thus, oxygen administration is a critical refinement, which should always be considered in injectable KX. However, Iso generally presented a safer profile, demonstrated by the fast induction and recovery times and the absence of mortality. Moreover, Iso also showed the lowest number of artifacts in immune cell activation and motility. These observations have clear implications for animal welfare and the refinement of immune studies, particularly for those involving intravital microscopy examinations, which require long-term anesthesia. This study type is essential in different fields, as cellular motility is a powerful indication of cellular activation^46^. Thus, our results contribute to understanding which protocol might be more appropriate to limit the generation of inaccurate results. Future research efforts should aim to elucidate the mechanisms underlying anesthesia-induced changes in cellular motility and develop strategies to mitigate these effects in IVM studies, such as multimodal balanced anesthesia.

## Methods

### Mice

Charles River Laboratories provided 6–8-week-old C57BL/6J mice, used in all the experiments. To evaluate immune cell motility, we used 6–12-week-old C57BL/6-Tg(UBC-GFP)30Scha/J (UBC-GFP) and Tg(CAG- ECFP)CK6Nagy/J (CK6-ECFP) mice, originally acquired from Jackson Laboratory (stock numbers 004353 and 003773, respectively) and bred in-house. The Institute for Research in Biomedicine (IRB) hosted animal experiments in facilities defined as specific pathogen-free, according to the FELASA guidelines. Mice were housed in individually ventilated cages under a controlled 12-h light/dark cycle, room temperature of 20–24 °C and relative humidity of 30–70%. Water and food were provided ad libitum. Animal caretakers, researchers and veterinarians checked the health of the animals daily. Euthanasia was always performed by cervical dislocation under deep anesthesia (Iso 5% for 5 min), followed by immediate organ collection. Equal numbers of males and females were assigned to experimental groups through a statistical randomization process. This was ensured through a blinded process, assigning each animal an ID and randomly sorting the IDs into experimental groups using dedicated software (R: A Language and Environment for Statistical Computing, R Core Team, R Foundation for Statistical Computing) or by an external blinded operator. Power calculation per groups size determination, performed using R software, was estimated to obtain >80% statistical power, starting from preliminary data previously collected in our laboratory in an independent experiment using similar techniques and setting the effect size (eta squared) at 15%. Each experiment was designed to extract more than a single outcome and minimize the number of repetitions of experiments. Therefore, for each group of outcomes, we set the group size according to the outcome requiring the highest number of replicates. Following this approach, the group sizes were *n* = 6 for Figs. 1 and 4 and Supplementary Figs. 1c,d, 4 and 5; *n* = 3 for Figs. 2 and 5 and Supplementary Figs. 1a,b and 2; and *n* = 6 per replicate, including three technical replicates (total *n* = 18) for Fig. 3 and Supplementary Fig. 3. Control animals, that is, not receiving anesthesia, were used only in Figs. 3 and 4. Positive controls, named ‘control’, received vaccination and followed the same group size as the experimental groups, while technical controls, whose average values are indicated with dashed red lines, did not receive either anesthesia or vaccination, and they were used with *n* = 3. The induction time (Fig. 1b) of one Iso-anesthetized animal was accidentally not registered. However, this did not affect the statistical analysis, given the extremely low standard deviation in this group, which is then composed of five replicates. In addition, in Fig. 1b,c, the KXO_2_ group is composed of seven replicates. This derives from testing a higher redosing in one extra animal, in a small independent pilot test. This test was unsuccessful because the animal died and was not included in the study. However, to maximize our data, we included the induction time and nonsurgical tolerance collected from this animal, which were comparable to the other animals in the same group, as the induction protocol was identical. However, we excluded this animal from the analysis of all other parameters (SpO_2_, HR, RR and mortality). Finally, in Fig. 1f, the numbers of the KXO_2_ group did not reach *n* = 6 because one animal died during the recovery phase, as indicated by the mortality rate in Fig. 1e. Therefore, the total number of animals used for this study is 115. All animal procedures and experiments were in accordance with the Swiss Federal Veterinary Service guidelines and authorized by the institutional committee (Commissione cantonale per gli esperimenti sugli animali) of the Cantonal Veterinary Office, with authorization numbers TI 28/17 and TI 104/24.

### Anesthetic protocols

The anesthetic protocols under investigation are shown in Fig. 1a, and the adaptations of analgesia and experimental design are described individually for each experiment through explanatory graphics in the corresponding figures. In general, for anesthesia maintenance, we administered the minimum concentration that guaranteed the absence of withdrawal reflexes in both fore and hind limbs during the procedures. The optimal KX concentration was tested as shown in Supplementary Fig. 1. In brief, we injected intraperitoneally (i.p.) 100/10 mg/kg of KX and maintained surgical anesthesia with additional administrations of 50/5 mg/kg at 30 min and 25/2.5 mg/kg at 60 min and 90 min after induction. In survival experiments (Fig. 1), all mice received i.p. BPP 30 min before the end of the procedure, at 0.1 (for KX and KXO_2_) or 0.05 mg/kg (for Iso), as requested by the authorities for nonterminal procedures. In all surgical experiments (Figs. 1 and 5) the Iso protocol was implemented by preemptive analgesia with 0.1 mg/kg BPP injected i.p. 30 min before the experiment. For the assessment of the effect of analgesia on the immune system (Supplementary Fig. 5), Iso was used either alone or in combination with BPP (0.1 mg/kg i.p. 30 min before the experiment) or with Lido (10 mg/kg subcutaneously (s.c.) 5 min before the experiment), as detailed in each figure and corresponding text. The KXO_2_ protocol differed from KX only in the additional administration of oxygen (FiO_2_ = 1, 1 l/min) in the induction box during induction and with a face mask during maintenance. In the Iso group, anesthesia was induced with 5% Iso in an induction box. Once surgical tolerance was reached, the anesthesia was continued with a face mask, and the Iso concentration decreased to 3% for the first 30 min and to 2% from 30 min to the end of the procedure. Oxygen drove gas for Iso administration (FiO_2_ = 1, 1 l/min). In all the protocols, induction took place in a preheated box, and using a heating plate, we maintained the body temperature constant in the range of 37–38.5 °C for the whole duration of the experiments. To ensure this, we measured body temperature using a rectal probe integrated into a closed-loop system with the warming pad.

### Anesthesia monitoring

For the evaluation of the mouse vital parameters, we induced anesthesia, and when mice reached surgical tolerance, they underwent surgery using the model of the exposure of the pLN for 2P-IVM, detailed below. At the end of the surgery, instead of performing microscopy, anesthesia was maintained to record vital parameters every 5 min for 2 h using a rodent monitor (Rodent Surgical Monitor+, Indus Instruments). For the duration of the experiments, we measured the following parameters: (1) induction time, defined as the time interval between drug administration (intraperitoneal injection of KX or exposure to 5% Iso) and the loss of righting reflex; (2) nonsurgical tolerance, defined as the time interval between the loss of righting reflex and the loss of fore and hind limb pedal reflex; (3) recovery time, as the interval between the regain of pedal reflex and the first forward movement; (4) mortality, as the percentage of mice that died during anesthesia or did not regain pedal reflex within 120 min after anesthesia discontinuation in relation to the total number of mice under study; (5) peripheral capillary SpO_2_, HR and RR, measured with the Rodent Surgical Monitor+ (Indus Instruments). Anesthetic depth was checked by the presence of pedal reflex, lid reflex and whisker movements every 5 min during the 2 h of anesthesia. At the end of the recording time, anesthesia administration was interrupted, and animal recovery was monitored for 2 h. Animals were euthanized as detailed above when they recovered walking capacity or after 2 h from the interruption of anesthesia for mice unable to recover.

### Flow cytometry of immune cells and inflammatory proteins

To evaluate the effect of different anesthetics on the inflammatory response, mice received influenza vaccination by injection of UV-inactivated influenza A/PR/8/34 (UV-PR8) virus strain. Virus production and UV inactivation were performed as described before^38^. In brief, the PR8 virus grew for 2 days in the allantoic cavity of 10-day embryonated chicken eggs. Then, we collected the allantoic fluid, removed cellular debris by centrifugation at 3,000 rpm for 30 min and purified the virus twice in a discontinuous sucrose gradient at 25,000 rpm for 90 min. To determine virus concentration, we performed a standard plaque assay. UV exposure for 15 min at a 15-cm distance inactivated the virus, which was then stored at −80 °C. A total of 10^7^ plaque-forming units of UV-PR8 were injected s.c. in both the mouse footpads in a volume of 10 μl sterile saline phosphate-buffered saline (PBS; Sigma-Aldrich). The injections were performed under Iso anesthesia within a 1-min timeframe. Monitoring the full recovery of mice from anesthesia guaranteed that no mice showed any side effects derived from anesthesia or vaccination. Two hours after UV-PR8 injection, anesthesia was induced, according to the assigned protocol, and maintained for 2 h, before euthanizing animals and collecting the right pLN for flow cytometry of the immune cells and the left pLN for cytokine and chemokine analysis.

For flow cytometry of the immune cells, we processed the pLN as previously described^48^. In brief, we physically disrupted the samples using tweezers and digested them for 10 min at 37 °C in an enzyme mixture of DNAse I (0.28 mg/ml, Amresco), Dispase (1 U/ml, Corning) and Collagenase P (0.5 mg/ml, Roche) in calcium- and magnesium-free PBS. The reaction was stopped with a solution of 2 mM EDTA (Sigma-Aldrich) and 2% heat-inactivated filter-sterilized fetal bovine serum (Thermo Fisher Scientific) in PBS. Then, the Fc receptors were blocked using an αCD16/32 antibody (93, BioLegend), and the surface of cells was stained for 30 min at 4 °C in a dark room using Zombie Aqua, αI-A/I-E – Pacific Blue (M5/114.15.2), αCD11b – Brilliant Violet 785 (M1/70), αCD11c – Brilliant Violet 711 (N418), αCD169 – PE (3D6.112), αF4/80 – Alexa Fluor 488 (BM8), αGr-1 – APC/Cy7 (RB6-8C5), αLy6G – Alexa Fluor 647 (1A8), αNK1.1 – PerCP/Cy5.5 (PK136), αCD3 – PE/Cy7 (17A2) and αB220 – Brilliant Violet 605 (RA3-6B2). All the antibodies were purchased from BioLegend, except αNK1.1, which was acquired from eBiosciences.

To evaluate the concentration of cytokines and chemokines, we applied two LEGENDPlex assays (Mouse Proinflammatory Chemokine Panel and Mouse Inflammation Panel, BioLegend) according to the instructions of the manufacturer. In brief, we carefully disrupted the pLN in cold PBS, minimizing cell rupture. After centrifugation at 1,500 rpm for 5 min we collected the supernatant and used it for the assay. For the acquisition of the number of immune cell and the inflammatory protein concentrations, we used an LSRFortessa (BD Bisociences). Data were then analyzed using the FlowJo software (BD Biosciences) or the LEGENDPlex software (BioLegend).

### Surgical preparation for intravital microscopy

For 2P-IVM of the immune cells, we adoptively transferred fluorescently labeled B cells, T cells and neutrophils from donor mice. B and T cells were isolated from the spleens of donor C57BL/6J and UBC-GFP mice, respectively. After mechanical tweezer and scissor disaggregation in a solution of 2 mM EDTA (Sigma-Aldrich) and 2% heat-inactivated filter-sterilized fetal bovine serum (Thermo Fisher Scientific) in PBS, we filtered the samples in a 40 µm nylon cell strainer (Cornig) and removed red blood cells using the ACK Lysing Buffer (Thermo Fisher Scientific). Then, we applied the CD19 MicroBeads for Mouse (Miltenyi) and the Pan T Cells Isolation Kit II for Mouse (Miltenyi) to isolate B and T cells, respectively. B cells were then labeled using the CellTracker Orange CMTMR dye (Thermo Fisher Scientific), following the manufacturer’s protocol. A mixture of 10 million B cells and 7 million T cells per mouse were injected intravenously (i.v.) in the tail vein of recipient C57BL/6J mice 12–18 h before imaging. Neutrophils were then isolated from the bone marrow of CK6-ECFP mice by collection and flushing of the femurs and tibias with cold, sterile PBS. Then, neutrophils were purified using the Neutrophils Isolation Kit for Mouse (Miltenyi) and injected (5 million) i.v. in the tail vein of recipient mice 2 h before imaging. At the same time, we injected s.c. in the footpad the influenza vaccine and the αCD169 – Alexa Fluor 647 (3D6.112) antibody to stain macrophages. For intravenous and subcutaneous injections, animals were briefly (<1 min) anesthetized with Iso 5%. After 2 h, we performed 2P-IVM by surgical exposure of the pLN as previously described^49^. In brief, we anesthetized mice according to the assigned protocol and positioned them on a customized surgical board. After fixing the tail and the leg, we dissected the skin and the subcutaneous fat in the popliteal fossa, taking care to avoid damage to blood or lymphatic vessels. Then, we submerged the exposed pLN in PBS and covered it with a glass coverslip. After 2 h of image acquisition, anesthetized animals were euthanized as previously described.

### 2P-IVM data generation and analysis

For deep in vivo tissue imaging, we used a customized two-photon platform (TrimScope, LaVision BioTec), with a set of two tunable Ti:Sa lasers (Chamaleon Ultra I,Chamaleon Ultra II, Coherent) and an optical parametric oscillator (OPO) that emits in the range of 1,010–1,340 nm (Chamaleon CompactOPO, Coherent), with output wavelengths in the range of 690–1,080 nm. Two-photon micrographs were acquired with 800 Hz scanning frequency, 520 × 520 pixels, a field of view of 278 × 278 µm^2^ and a line average 1. We set the first Ti:Sa laser to 830 nm to excite the second-harmonic generation from collagen, the second Ti:Sa laser to 925 nm to excite GFP and RFP, and the OPO laser to 1,100 nm to excite far-red fluorescence. We acquired a full *Z* stack of 40 µm every 30 s for a total of 30 min, repeating the acquisition four times for a total of 2 h of recordings. To analyze immune cell motion quantitatively, we used manual tracking (Imaris 9.1.2, Bitplane). For the statistical analysis of cell migration, we considered only track durations greater than 5 min.

### Statistical analyses

All raw data were analyzed, processed and presented using GraphPad Prism 10.2.0 (GraphPad Software). First, we applied the Shapiro–Wilk normality test to analyze the distribution of data. Then, we compared means among groups using one-way analysis of variance or unpaired *t*-test for data with normal distribution and the nonparametric Kruskal–Wallis or Mann–Whitney test for groups that did not present a normal distribution. Data normally distributed in all the experimental groups include the results represented in the following figures: Fig. 1c,d and Supplementary Figs. 1c,d, 3c, 4c,d,g,i,k,r and 5b–d,f,h–j. Data not normally distributed include the results showed in the following figures: Figs. 1b,f and 2d,e and Supplementary Figs. 2a–c, 3a–f, 4a–f,h,j,l–q,s and 5b–g. *P* values are indicated in the figures as *P ≤ 0.05, **P < 0.01, ***P < 0.001, ****P < 0.0001.

### Reporting summary

Further information on research design is available in the Nature Portfolio Reporting Summary linked to this article.

## Online content

Any methods, additional references, Nature Portfolio reporting summaries, source data, extended data, supplementary information, acknowledgements, peer review information; details of author contributions and competing interests; and statements of data and code availability are available at 10.1038/s41684-025-01614-4.

## Supplementary information

Supplementary Information Supplementary Figs. 1–5.

Reporting Summary

Supplementary Movie 1 Neutrophils.

Supplementary Movie 2 T cells.

Supplementary Movie 3 B cells.

## Data Availability

The data that support the findings of this study are available from the corresponding author upon request. Intravital microscopy datasets are available via Immunemap at https://www.immunemap.org/.
