# Supplementary material for: Impact of prolonged isoflurane or ketamine–xylazine anesthesia with or without buprenorphine and oxygen on mouse vitals and immune responses
Source: Lab Anim (NY). 2025 Sep 18;54(10):270–7. doi: 10.1038/s41684-025-01614-4 (PMC12484078; doi:10.1038/s41684-025-01614-4)
Supplement: Supplementary file 1 — Supplementary Figs. 1–5. [file 41684_2025_1614_MOESM1_ESM.pdf]

lab animal

Supplementary information

https://doi.org/10.1038/s41684-025-01614-4

Impact of prolonged isoflurane or ketamine–xylazine anesthesia with or without buprenorphine and oxygen on mouse vitals and immune responses

In the format provided by the authors and unedited

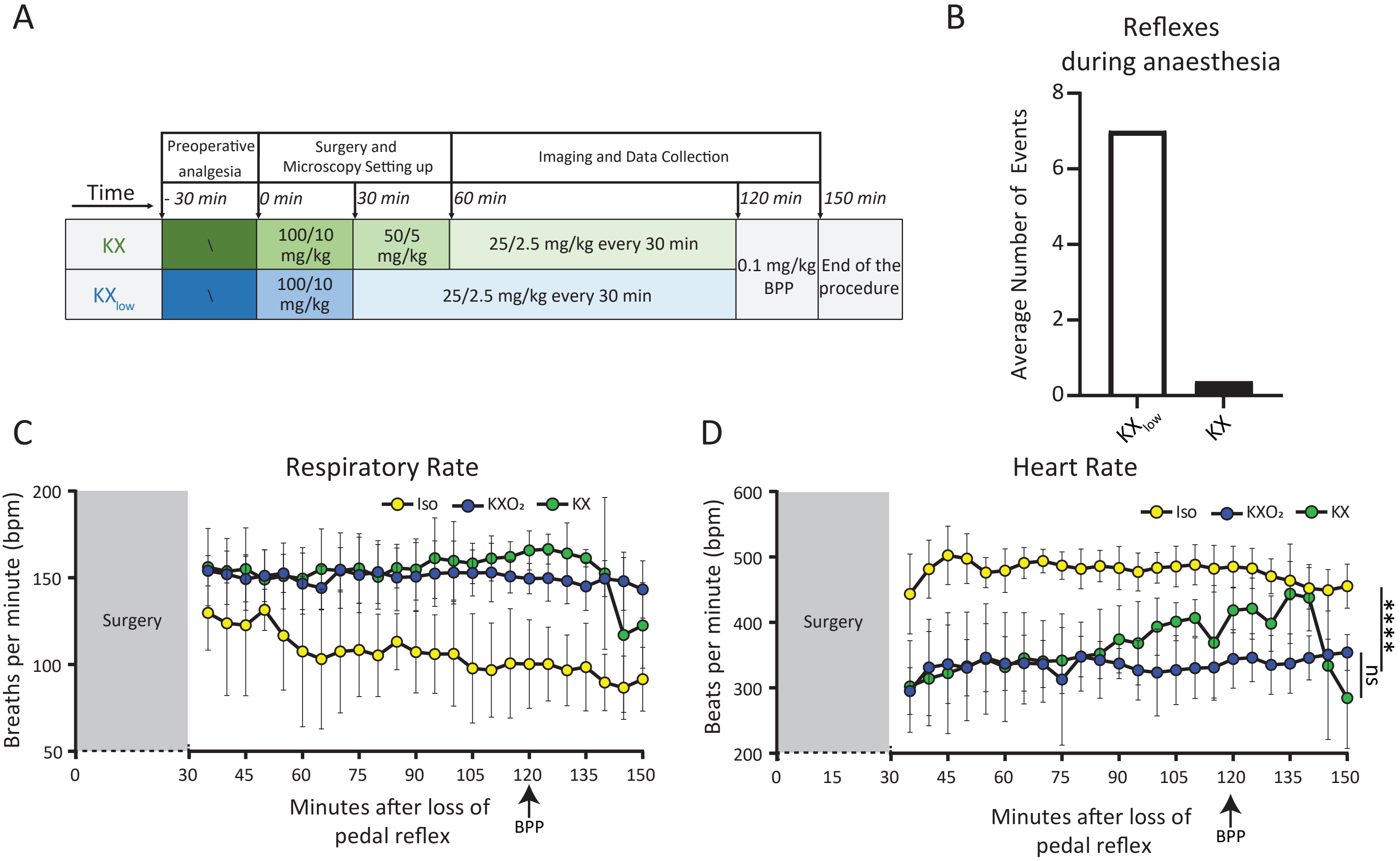

Supplementary Figure 1. *(A) Schematic representation of two KX redosing protocols. (B) Average number of reflexes observed during surgical tolerance. (C) Respiratory rate and (D) heat rate in the three groups over time. BPP shows the time of injection of buprenorphine in all the three goups. Circles represent mean values per group, and lines indicate standard deviation.*

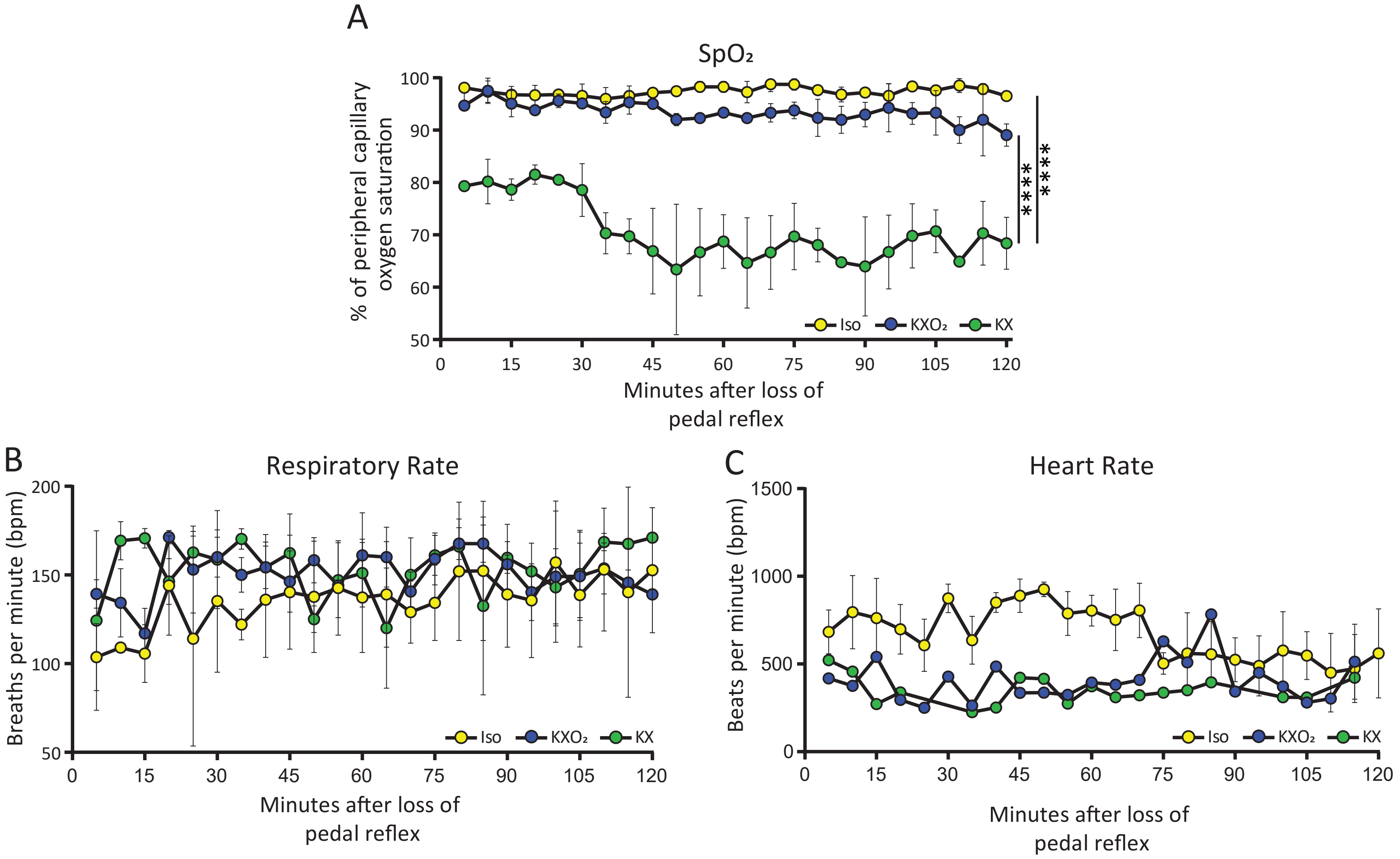

Supplementary Figure 2. *(A) Oxygen Saturation, (B) Respiratory Rate, and (C) Heat Rate in the three protocols, without BPP administration, over time. Oxygen Saturation is indicated in percentage, while Respiratory Rate and Heart Rate are described in beats per minute (bpm). Circles represent mean values per group, and lines indicate standard deviation.*

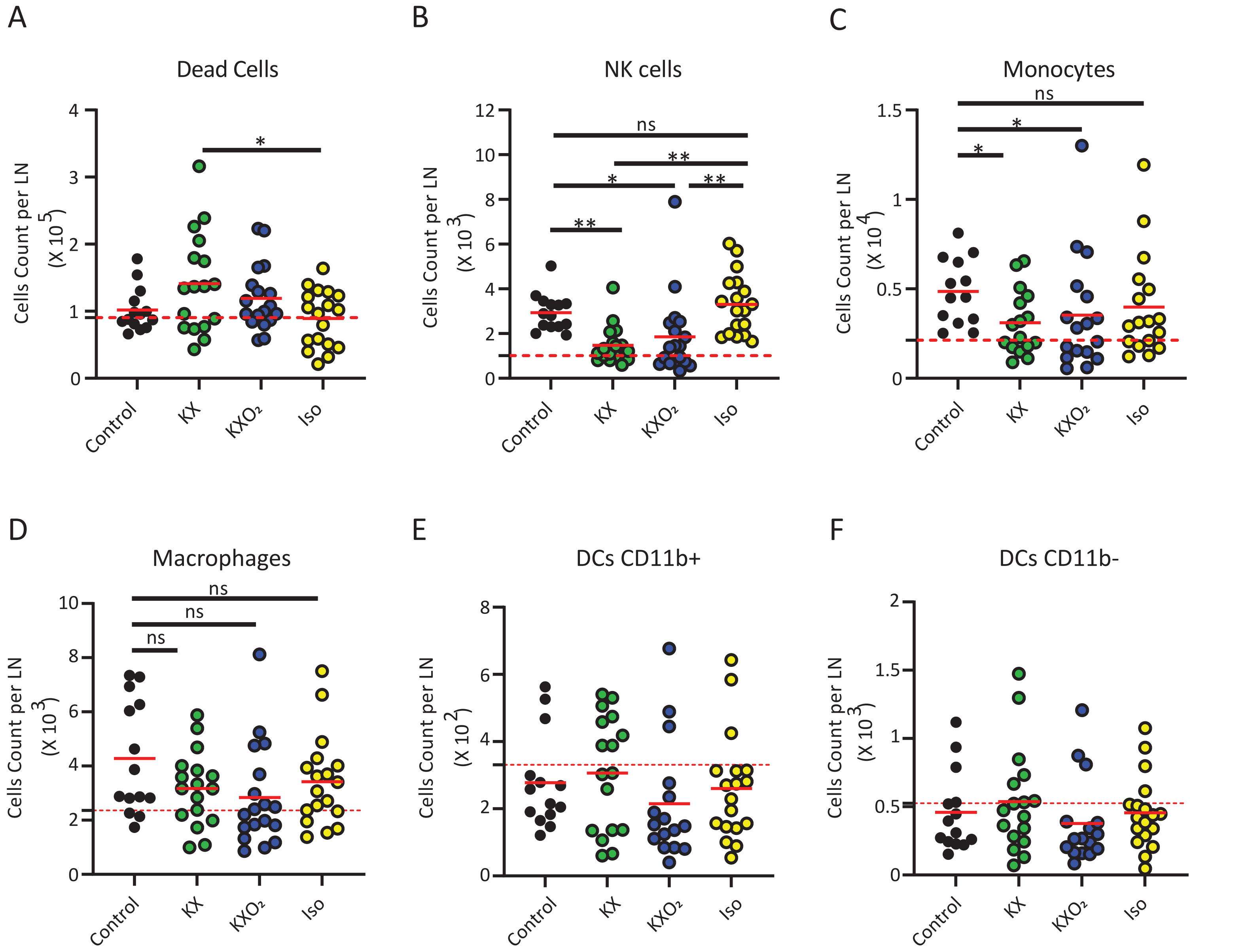

Supplementary Figure 3. *Flow cytometric quantification of the number of total (A) dead cells, (B) NK cells, (C) monocytes, (D) macrophages, (E) CD11b positive activated dendritic cells, and (F) CD11b negative dendritic cells in the pLN of vaccinated and anesthetized mice in comparison to non anesthetized controls. Circles and red lines represent individual and mean values, respectively.*

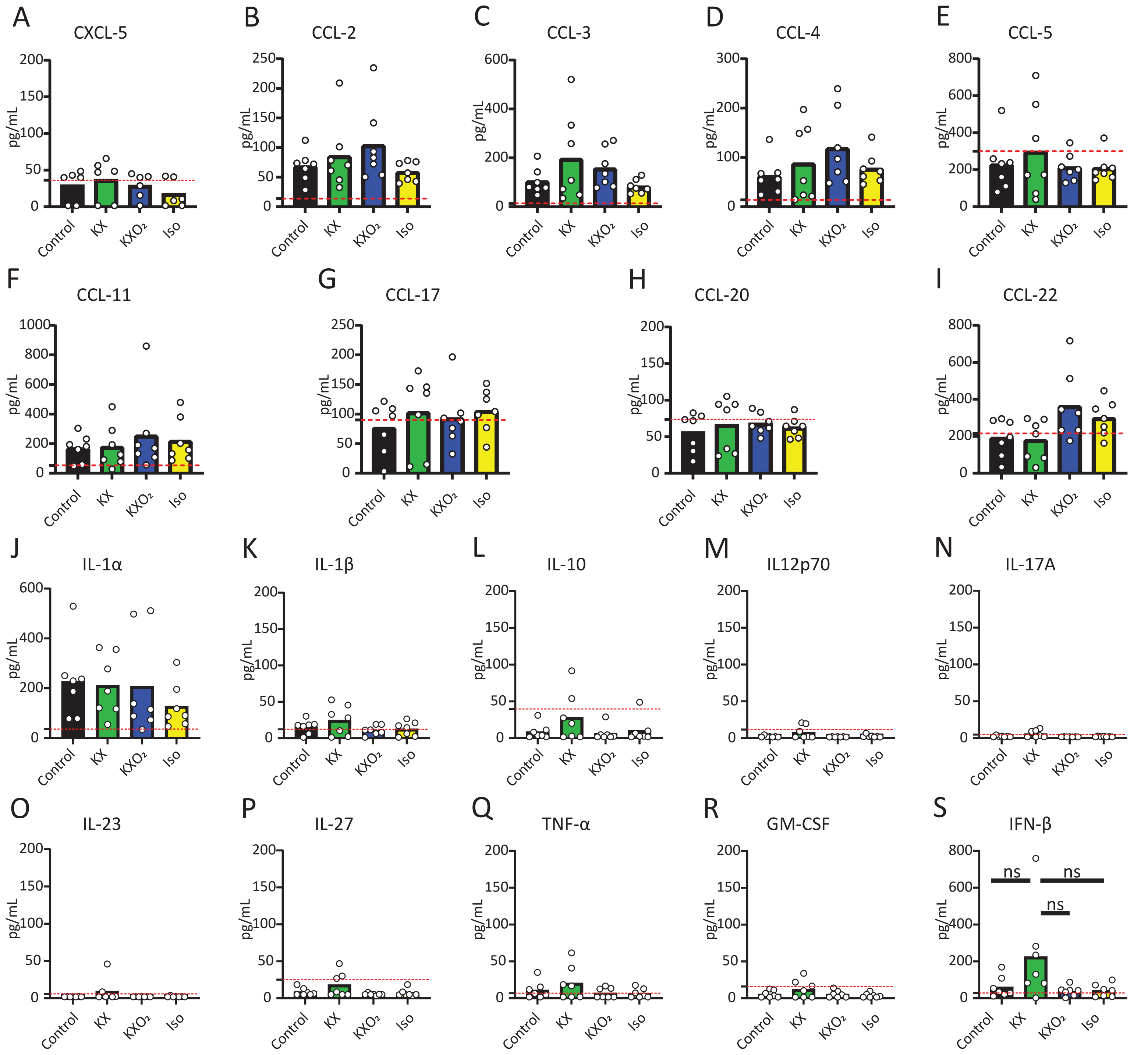

Supplementary Figure 4. *Concentration of different inflammatory cytokines and chemokines in the pLN supernatant of vaccinated anesthetized mice in comparison to not anesthetized controls. Panels show (A) CXCL-5, (B) CCL-2, (C) CCL-3, (D) CCL-4, (E) CCL-5, (F) CCL-11, (G) CCL-17, (H) CCL-20, (I) CCL-22, (J) interleukin-1α, (K) interleukin-1β, (L) interleukin-10, (M) interleukin-12p70, (N) interleukin-17A, (O) interleukin-23, (P) interleukin-27, (Q) tumor necrosis factor-α, (R) granulocyte-macrophage colony stimulating factor, and (S) interferon-β. Circles and bars show individual and average values, respectively. The red dashed line indicates the mean value in non-vaccinated animals.*

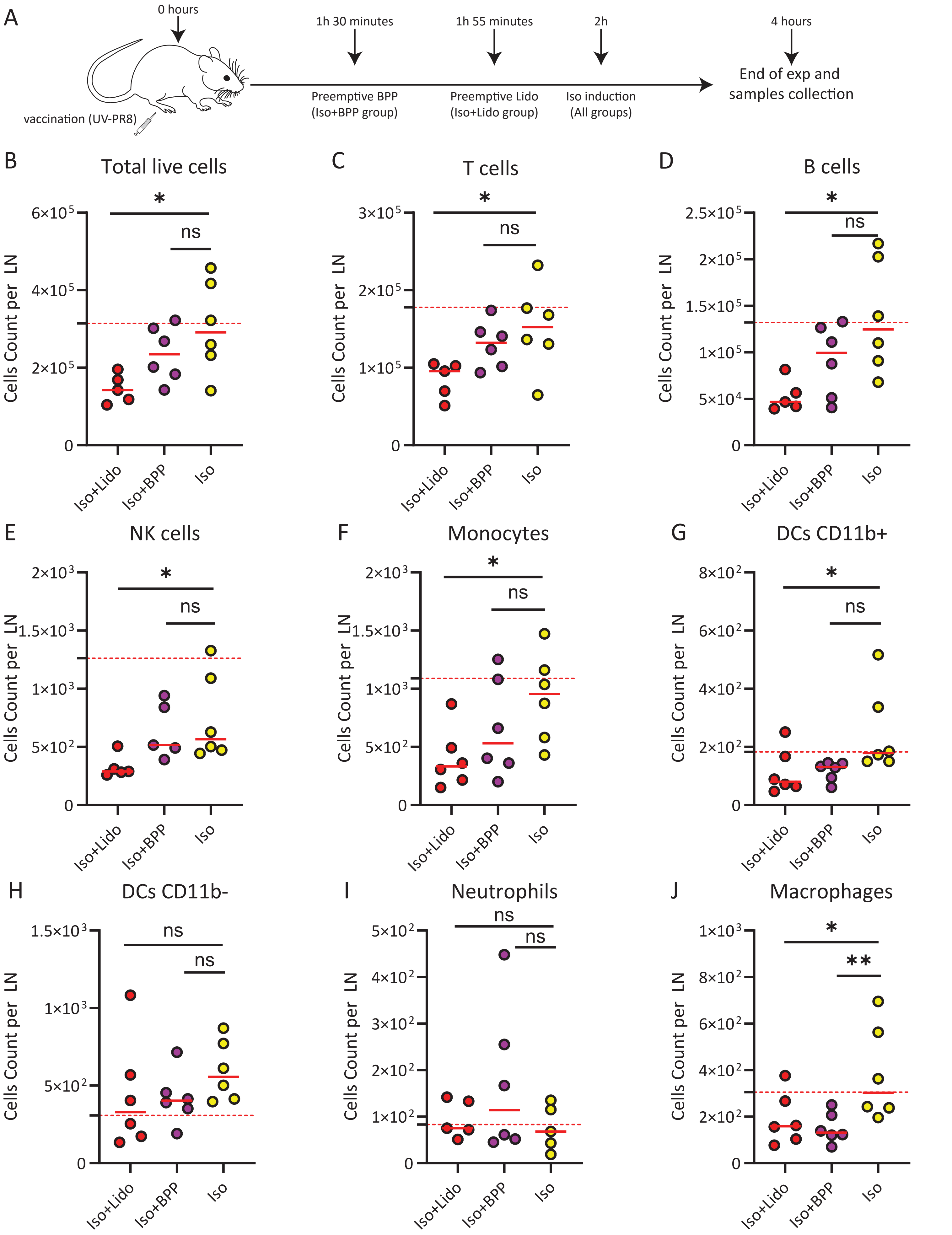

Supplementary Figure 5. *(A) Experimental design showing timing of each analgesic protocol in the 3 Iso groups. BPP was administered ip at 0.1 mg/kg 30 minutes before induction of general anesthesia. Lidocaine was injected sc at 10 mg/kg 5 minutes before induction of anesthesia. Flow cytometric quantification of the number of total (B) live cells, (C) T cells, (D) B cells, (E) NK cells, (F) Monocytes, (G) CD11b positive dendritic cells, (H) CD11b negative dendritic cells, (I) Neutrophils, and (J) Macrophages in the pLN of mice vaccinated and anesthetized with Iso only (yellow), Iso + preemptive BPP (violet), or Iso + preemptive Lidocaine (Red), in comparison to non anesthetized controls (red dashed line). Circles and red lines represent individual and mean values, respectively.*
