# Supplementary material for: Impact of prolonged isoflurane or ketamine–xylazine anesthesia with or without buprenorphine and oxygen on mouse vitals and immune responses
Source: Lab Anim (NY). 2025 Sep 18;54(10):270–7. doi: 10.1038/s41684-025-01614-4 (PMC12484078; doi:10.1038/s41684-025-01614-4)
Supplement: Supplementary file 2 — Reporting Summary [file 41684_2025_1614_MOESM2_ESM.pdf]

nature portfolio

Corresponding author(s): DBPR, LABAN-A01981A

Last updated by author(s): Jan 28, 2025

Reporting Summary

Nature Portfolio wishes to improve the reproducibility of the work that we publish. This form provides structure for consistency and transparency in reporting. For further information on Nature Portfolio policies, see our Editorial Policies and the Editorial Policy Checklist.

Statistics

For all statistical analyses, confirm that the following items are present in the figure legend, table legend, main text, or Methods section.

n/a	Confirmed	
☐	☒	The exact sample size (n) for each experimental group/condition, given as a discrete number and unit of measurement
☐	☒	A statement on whether measurements were taken from distinct samples or whether the same sample was measured repeatedly
☐	☒	The statistical test(s) used AND whether they are one- or two-sided<br>*Only common tests should be described solely by name; describe more complex techniques in the Methods section.*
☐	☒	A description of all covariates tested
☐	☒	A description of any assumptions or corrections, such as tests of normality and adjustment for multiple comparisons
☐	☒	A full description of the statistical parameters including central tendency (e.g. means) or other basic estimates (e.g. regression coefficient) AND variation (e.g. standard deviation) or associated estimates of uncertainty (e.g. confidence intervals)
☐	☒	For null hypothesis testing, the test statistic (e.g. F, t, r) with confidence intervals, effect sizes, degrees of freedom and P value noted<br>*Give P values as exact values whenever suitable.*
☒	☐	For Bayesian analysis, information on the choice of priors and Markov chain Monte Carlo settings
☒	☐	For hierarchical and complex designs, identification of the appropriate level for tests and full reporting of outcomes
☐	☒	Estimates of effect sizes (e.g. Cohen's d, Pearson's r), indicating how they were calculated

Our web collection on statistics for biologists contains articles on many of the points above.

Software and code

Policy information about availability of computer code

Data collection	No software was used.
Data analysis	To analyze immune cells motion quantitatively, we employed Imaris 9.1.2, Bitplane.

For manuscripts utilizing custom algorithms or software that are central to the research but not yet described in published literature, software must be made available to editors and reviewers. We strongly encourage code deposition in a community repository (e.g. GitHub). See the Nature Portfolio guidelines for submitting code & software for further information.

Data

Policy information about availability of data

All manuscripts must include a data availability statement. This statement should provide the following information, where applicable:

- Accession codes, unique identifiers, or web links for publicly available datasets
- A description of any restrictions on data availability
- For clinical datasets or third party data, please ensure that the statement adheres to our policy

All data supporting the findings of this study are available within the paper and its Supplementary Information.

Field-specific reporting

Please select the one below that is the best fit for your research. If you are not sure, read the appropriate sections before making your selection.

☒ Life sciences ☐ Behavioural & social sciences ☐ Ecological, evolutionary & environmental sciences

For a reference copy of the document with all sections, see nature.com/documents/nr-reporting-summary-flat.pdf

Life sciences study design

All studies must disclose on these points even when the disclosure is negative.

Sample size	Power calculation per groups size determination, performed by using R software, estimated 6 animals per group to obtain > 95 % statistical power.
Data exclusions	No data have been excluded from the study.
Replication	Replicates in each experiment represent individual animals, thus being biological and technical replicates. In figure 4, individual points represent cells, pooled from three animals (independent experiments). Each experiment has been performed at least three times to confirm the alignment of the obtained data. Data from replicate experiments have been pooled in the graphs.
Randomization	Animals were assigned to an experimental group through a blind process, by receiving an ID and being then sorted casually to an experimental group by using a dedicated software (R: A Language and Environment for Statistical Computing, R Core Team, R Foundation for Statistical Computing, Vienna, Austria) or by an external blind operator.
Blinding	Animals were assigned to an experimental group through a blind process, by receiving an ID and being then sorted casually to an experimental group by using a dedicated software (R: A Language and Environment for Statistical Computing, R Core Team, R Foundation for Statistical Computing, Vienna, Austria) or by an external blind operator.

Reporting for specific materials, systems and methods

We require information from authors about some types of materials, experimental systems and methods used in many studies. Here, indicate whether each material, system or method listed is relevant to your study. If you are not sure if a list item applies to your research, read the appropriate section before selecting a response.

Materials & experimental systems

n/a	Involved in the study	
☐	☒	Antibodies
☒	☐	Eukaryotic cell lines
☒	☐	Palaeontology and archaeology
☐	☒	Animals and other organisms
☒	☐	Human research participants
☒	☐	Clinical data
☒	☐	Dual use research of concern

Methods

n/a	Involved in the study	
☒	☐	ChIP-seq
☐	☒	Flow cytometry
☒	☐	MRI-based neuroimaging

Antibodies

Antibodies used	Fc receptors were blocked using an αCD16/32 antibody (93, BioLegend), and the surface of cells was stained for 30 minutes at 4°C in a dark room using Zombie Aqua, αI-A/I-E – Pacific Blue (M5/114.15.2), αCD11b – Brilliant Violet 785 (M1/70), αCD11c – Brilliant Violet 711 (N418), αCD169 – PE (3D6.112), αF4/80 – Alexa Fluor 488 (BM8), αGr-1 – APC/Cy7 (RB6-8C5), αLy6G – Alexa Fluor 647 (1A8), αNK1.1 – PerCP/Cy5.5 (PK136), αCD3 – PE/Cy7 (17A2), αB220 – Brilliant Violet 605 (RA3-6B2). All the antibodies were purchased from BioLegend, with the exception of αNK1.1, acquired from eBiosciences.
Validation	All antibodies have been previously validated by the commercial provider.

Animals and other organisms

Policy information about studies involving animals; ARRIVE guidelines recommended for reporting animal research

Laboratory animals	Charles River Laboratories provided 6-8 weeks C57BL/6J mice, used in all the experiments. To evaluate immune cells motility we used 6-12 weeks C57BL/6-Tg(UBC-GFP)30Scha/J (UBC-GFP) and Tg(CAG-ECFP)CK6Nagy/J (CK6-ECFP) mice, originally acquired from Jackson Laboratory (respective Stock Numbers 004353 and 003773) and bred in-house. Equal numbers of males and females (3 + 3) were assigned to experimental groups.

Wild animals | The study did not involve wild animals.

Field-collected samples | The study did not involve data collected from the field.

Ethics oversight | All animal procedures and experiments were in accordance with the Swiss Federal Veterinary Service guidelines and authorized by the institutional committee (Commissione cantonale per gli esperimenti sugli animali) of the Cantonal Veterinary Office, with authorization number TI28/17.

Note that full information on the approval of the study protocol must also be provided in the manuscript.

Flow Cytometry

Plots

Confirm that:

- [ ] The axis labels state the marker and fluorochrome used (e.g. CD4-FITC).
- [x] The axis scales are clearly visible. Include numbers along axes only for bottom left plot of group (a 'group' is an analysis of identical markers).
- [ ] All plots are contour plots with outliers or pseudocolor plots.
- [x] A numerical value for number of cells or percentage (with statistics) is provided.

Methodology

Sample preparation | For flow cytometry of the immune cells we physically disrupted the samples using tweezers and digested them for 10 minutes at 37°C in an enzyme mixture of DNAse I (0.28 mg/mL, Amresco), Dispase (1 U/mL, Corning) and Collagenase P (0.5 mg/mL, Roche) in calcium- and magnesium-free PBS. The reaction was stopped with a solution of 2mM EDTA (Sigma-Aldrich) and 2% heat-inactivated filter-sterilized fetal bovine serum (Thermo Fisher Scientific) in PBS. Then, the Fc receptors were blocked using an αCD16/32 antibody (93, BioLegend), and the surface of cells was stained for 30 minutes at 4°C in a dark room using Zombie Aqua, αI-A/I-E – Pacific Blue (M5/114.15.2), αCD11b – Brilliant Violet 785 (M1/70), αCD11c – Brilliant Violet 711 (N418), αCD169 – PE (3D6.112), αF4/80 – Alexa Fluor 488 (BM8), αGr-1 – APC/Cy7 (RB6-8C5), αLy6G – Alexa Fluor 647 (1A8), αNK1.1 – PerCP/Cy5.5 (PK136), αCD3 – PE/Cy7 (17A2), αB220 – Brilliant Violet 605 (RA3-6B2). All the antibodies were purchased from BioLegend, with the exception of αNK1.1, acquired from eBiosciences.
To evaluate the concentration of cytokines and chemokines, we applied two LEGENDPlexTM assays (Mouse Proinflammatory Chemokine Panel and Mouse Inflammation Panel; BioLegend) according to the instructions of the manufacturer. Briefly, we carefully disrupted the pLN in cold PBS, minimizing cell rupture. After centrifugation at 1500 rpm for 5 minutes we collected the supernatant and use it for the assay.

Instrument | For the acquisition of the immune cells numbers and the inflammatory proteins concentrations we used a LSRFortessaTM (BD Bisociences).

Software | Data were then analyzed using the FlowJo software (BD Biosciences) or the LEGENDPlex software (BioLegend).

Cell population abundance | Cells numbers are represented in the relative graphs (Figure 2 and Supp Figure 2).

Gating strategy | We first gated on SSC-A and FSC-A to exclude debris and on FSC-A and FSC-H to select singlets. We next eliminated Live-Dead + dead cells and selected CD3+ B220- T cells and CD3-B220+ B cells. NK cells were gated as CD3- B220- NK1.1+. Neutrophils and monocytes were selected as CD3- B220- Gr1int Ly6G+ and CD3- B220- Gr1+ Ly6G-, respectively. Macrophages and DCs were gated as CD3- B220- MHC II+ CD11b+ CD11cintlow F4/80+ and CD3- B220- MHC II+ CD11c+ F4/80-, respectively. Then, DCs were further divided into CD11b+ and CD11b-.

- [ ] Tick this box to confirm that a figure exemplifying the gating strategy is provided in the Supplementary Information.
